# Supplementary material for: Galactosylceramide Upregulates the Expression of the BCL2 Gene and Downregulates the Expression of TNFRSF1B and TNFRSF9 Genes, Acting as an Anti-Apoptotic Molecule in Breast Cancer Cells
Source: Cancers (Basel). 2024 Jan 17;16(2):389. doi: 10.3390/cancers16020389 (PMC10813928; doi:10.3390/cancers16020389)
Supplement: Supplementary file 1 [file cancers-16-00389-s001.zip › Caption for supplementary materials.pdf]

Supplementary Materials for Suchanski et al.

Galactosylceramide Upregulates the Expression of the BCL2 Gene and Downregulates the Expression of TNFRSF1B and TNFRSF9 Genes, Acting as an Ani-apoptotic Molecule in Breast Cancer Cells

Supplementary Tables

Table S1: List of primers used in this study.

Table S2: List of primers for apoptosis gene expression profiling by RT-qPCR array.

Figure legends

Figure S1: Scheme of UGT8-specific 20 nucleotide guide RNA (gRNA-TGTGATAGCTCATCTTTTAG) targeting exon 2 in *UGT8* gene and PCR products with mutation induced by CRISPR/Cas9. Protospacer-adjacent motif (PAM) sequence underlined. Arrows indicate the locations of PCR primers.

Figure S2: Proliferative potential of BC MDA-MB-231, MCF7, and T47D cells with different expression of GalCer. Cell cycle progression after 48 h in **(A)** parental MDA-MB-231, MDA-MB-231 transduced with vector alone (MDA.Δ.C), and MDA-MB-231 cell clones with knockout of *UGT8* gene (MDA.Δ.UGT8.1, and MDA.Δ.UGT8.4), **(B)** parental MCF7, MCF7 transduced with vector alone (MCF7.C), and MCF7 with overexpression of UGT8 (MCF7.UGT8) cells, **(C)** parental T47D, control T47D transduced with vector alone (T47D.C), and T47D cells with overexpression of UGT8 (T47D.UGT8). The number of cell nuclei in a given phase of the cell cycle was calculated by flow cytometry after staining with "FxCellcycle PI/RNAse "solution (Thermo Fisher Scientific). Data are presented as the average of three independent measurements (n=3).

Figure S3: Differentially expressed apoptosis-related genes in BC cellular models with different content of GalCer. Venn plot shows the common apoptotic genes (*BCL2*, *TNFRSF1*, and *TNFRSF9*) shared by BC cells with high (MDA.Δ.C, MCF7.UGT8, and T47D.UGT8) and low (MDA.Δ.UGT8.4, MCF7.C, and T47D.C). Arrows indicate an increase or decrease in gene expression levels.

Figure S4: cDNA transcriptional microarray analysis of apoptotic genes in human BC cell lines with a knockout of *UGT8* gene - MDA.Δ.UGT8.4 vs. MDA.Δ.C **(A)**, overexpression of *UGT8* gene - MCF7.UGT8 vs. MCF7.C **(B),** and T47D.UGT8 vs. T47D.C **(C)**. The relative expression levels for each gene are plotted against the same gene from the control group. The middle line shows a similar expression in both groups with three-fold change boundaries. Genes upregulated greater than three-fold in BC cells lie above the boundary line, and downregulated genes lie below the boundary line. Genes that are upregulated in BC cells with knockout of the *UGT8* gene and simultaneously down-regulated in BC cells with overexpression of the *UGT8* gene are indicated in the rectangle.

Figure S5: Sensitivity of BC MDA-MB-231 and MCF7 cells with different GalCer levels to apoptosis induced by DOX, grown in the presence of ABT-199 (a specific inhibitor of Bcl-2). **(A)** MDA.Δ.C - MDA-MB-231 transduced with vector alone and MDA.Δ.UGT8.4 - MDA-MB-231 cell clone with a knockout of *UGT8* gene; **(B)** MCF7.C- MCF7 transduced with vector alone and MCF7.UGT8 - MCF7 with overexpression of UGT8. The percentage of apoptotic cells was determined by flow cytometry using Annexin V and SYTOX Green stain.

Figure S6: Sensitivity of BC MDA-MB-231 and MCF7 cells with different GalCer levels to apoptosis incubated with TNFα. **(A)** MDA.Δ.C - MDA-MB-231 transduced with vector alone

and MDA.Δ.UGT8.4 - MDA-MB-231 cell clone with knock-out of *UGT8* gene; **(B)** MCF7.C - MCF7 transduced with vector alone and MCF7.UGT8 - MCF7 with overexpression of UGT8. The percentage of apoptotic cells was determined by flow cytometry using Annexin V and SYTOX Green stain.

Figure S7: Nucleotide sequences of *BCL2*, *TNFRSF9*, and *TNFRSF1B* promoters. Potential sequences for transcriptional factors present in all three promoters are boxed. Nucleotides are numbered with the translational initiation site designated as +1.

Figure S8: Expression of **(A)** CREB and **(B)** NFκB in MDA-MB-231 cells transduced with "empty" vector (MDA.Δ.C), MDA-MB-231 cell clones with knockout of the UGT8 gene (MDA.Δ.UGT8.4), MCF7 cells transduced with vector alone (MCF7.C), and MCF7 cells overexpressing UGT8 and GalCer (MCF7.UGT8). Western blotting was used to analyze CREB and NFκB expression in BC cell lines. GAPDH was used as the internal control.
