# Supplementary material for: Galactosylceramide Upregulates the Expression of the BCL2 Gene and Downregulates the Expression of TNFRSF1B and TNFRSF9 Genes, Acting as an Anti-Apoptotic Molecule in Breast Cancer Cells
Source: Cancers (Basel). 2024 Jan 17;16(2):389. doi: 10.3390/cancers16020389 (PMC10813928; doi:10.3390/cancers16020389)
Supplement: Supplementary file 1 [file cancers-16-00389-s001.zip › Table S1.pdf]

Table S1. List of primers used in this study, related to the experimental procedures

primer	species	application	annealing temp. (^oC)	primer sequence 5'-3'	primer length	reference
RT-forUGT8a	mouse	Real-time-PCR assay	55	CATGGTGTCCCTGTAGTGG	19	This work
RT-revUGT8a				GAGCCCTCTGCCGATAAC	18	
RT-forAKT-m	mouse		55	ACCACACCTTCTACAATGAGC	21	This work
RT-revAKT-m				GACAGCACAGCCTGGATGGC	20	
RT-forUGT8	human		55	CATGGTGTGCCTGTAGTGG	19	Owczarek et al., 2013
RT-revUGT8				GAGCCCTCTGACGGTAGC	18	
RT-forAKT	human		55	ACCACACCTTCTACAATGAGC	21	Suchanski et al., 2017
RT-revAKT				GATAGCACAGCCTGGATAGC	20	
RT-forTNFRSF1B	human		58	CACATGCCGGCTCAGAGAAT	20	This work
RT-revTNFRSF1B				AGCTGGGTGTATGTGCTGTC	20	
RT-forTNFRSF9	human		58	CTCACGCTCCGTTTCTCTGT	20	This work
RT-revTNFRSF9				GAAATCGGCAGCTACAGCCA	20	
RT-forBCL2	human		58	CTTTGAGTTCGGTGGGGTCA	20	This work
RT-revBCL2				GGGCCGTACAGTTCCACAAA	20	
forMluI-P-Bcl2	human	Promotor insertion into pGL3-Basic luciferase vector	55	CA**ACGCGT**GAGGGCTCTTTCTTTCTTCT<br>**MluI**	28	This work
revHindIII-P-Bcl2				GA**AAGCTT**AGCTGATTTGAAACTTCCCA<br>**HindIII**	28	
forMluI-P- TNFRSF9	human		55	CA**ACGCGT**CCTCCTTCCTCCCTCTGTC<br>**Mlu I**	27	This work
revHindIII-P-TNFRSF9				GA**AAGCTT**CCCCAGATGGACAGACCTT<br>**HindIII**	27	
forMluI-P-TNFRSF1B	human		55	CA**ACGCGT**ATGGTGGCCCGAGCCTGT<br>**Mlu I**	26	This work
revHindIII-P-TNFRSF1B				GA**AAGCTT**TGGGCCGCGCGAGTCA<br>**HindIII**	24	
forEcoRI-UGT8a	mouse	UGT8a-expressing vector construction	52	C**GAATTC**ATGAAGTCTTACACTCC<br>**EcoRI**	24	This work
revMluI-UGT8a				GA**ACGCGT**TCATCTCACCTTCTTTT<br>**MluI**	25	
forXhoI-UGT8	human	UGT8-expressing vector construction	52	C**CTCGAG**CGCCTCCATGAAGTCTTACACTCCATATT<br>**XhoI**	29	Suchanski et al., 2017
revBamHI-UGT8				GC**GGATCC**TCATTTCACTTTCTTTTCATGT<br>**BamhI**	22	
F1-exon2/UGT8	human	Genome sequencing after CRISPR-Cas9 editing	55	GCCGCCAATTATGTTTGAAAGC	22	This work
R1-exon2/UGT8				GGCTGGACCCATGAACCAAA	20	
