# Supplementary material for: Galactosylceramide Upregulates the Expression of the BCL2 Gene and Downregulates the Expression of TNFRSF1B and TNFRSF9 Genes, Acting as an Anti-Apoptotic Molecule in Breast Cancer Cells
Source: Cancers (Basel). 2024 Jan 17;16(2):389. doi: 10.3390/cancers16020389 (PMC10813928; doi:10.3390/cancers16020389)
Supplement: Supplementary file 1 [file cancers-16-00389-s001.zip › Table S2.pdf]

Table S2. List of primers used in apoptosis gene expression profiling with a RT-qPCR array

GenBank	Gen	Description	Type	Primers	Sequence 5'---3'	bp
NM_000594	TNF	Tumor necrosis factor	Pro-Apoptotic	TNF-F	ACTTTGGAGTGATCGGCCC	192
				TNF-R	CATTGGCCAGGAGGGCATT	
NM_003805	CRADD	CASP2 and RIPK1 domain containing adaptor with death domain		CRADD-F	GTTGCAGTTTTTCTCCCCCG	121
				CRADD-R	CCCTCCACCAATACCTCTGC	
NM_003824	FADD	Fas (TNFRSF6)-associated via death domain		FADD-F	CTGGAGCAGAACGACCTGG	196
				FADD-R	GCCTTCTCCAATCTTTCCCCA	
NM_005157	ABL1	C-abl oncogene 1, non-receptor tyrosine kinase		ABL1-F	TCCTCGTCCTCCAGCTGTTA	152
				ABL1-R	GCAACGAAAAGGTTGGGGTC	
NM_000546	p53	Tumor protein p53		p53-F	CCAACAACACCAGCTCCTCT	118
				p53-R	AAGGCCTCATTCAGCTCTCG	
NM_001279	CIDEA	Cell death-inducing DFFA-like effector a		CIDEA-F	CTCATCAGGCCCCTGACATT	199
				CIDEA-R	GCACCAGAGTGACCAGTCC	
NM_003879	CFLAR	CASP8 and FADD-like apoptosis regulator		CFLAR-F	CGGGAGGAGGTGTAGGAGAG	186
				CFLAR-R	ATCCTTTCCAGTGGGGGAGT	
NM_004938	DAPK1	Death-associated protein kinase 1		DAPK1-F	GCAGGTCTCCTTGCAAGACT	160
				DAPK1-R	GCAGCCCACACTTGAGAGAT	
NM_004322	BAD	BCL2-associated agonist of cell death		BAD-F	TCCGGAGGATGAGTGACGAG	128
				BAD-R	ATCCCACCAGGACTGGAAGA	
NM_001188	BAK1	BCL2-antagonist/killer 1		BAK1-F	GCAGGCTGATCCCGTCC	187
				BAK1-R	CCTTCAGCCTCCTGTTCCTG	
NM_004324	BAX	BCL2-associated X protein		BAX-F	CCCCCGAGAGGTCTTTTTCC	160
				BAX-R	TGTCCAGCCCATGATGGTTC	
NM_001196	BID	BH3 interacting domain death agonist		BID-F	GAAGCGGGTAGTCGACCG	165
				BID-R	ACCGTTGTTGACCTCACAGT	
NM_001197	BIK	BCL2-interacting killer (apoptosis-inducing)		BIK-F	GGAGGTTCTTGGCATGACTGA	133
				BIK-R	GCTCACGTCCATCTCGTCC	
NM_033292	CASP1	Caspase 1, apoptosis-related cysteine peptidase (interleukin 1, beta, convertase)		CASP1-F	TCCGTTATTCCGAAAGGGGC	191
				CASP1-R	ATAGCTGGGTTGTCCTGCAC	
NM_004346	CASP3	Caspase 3, apoptosis-related cysteine peptidase		CASP3-F	TGCATACTCCACAGCACCTG	154
				CASP3-R	TTCTGTTGCCACCTTTCGGT	
NM_001229	CASP9	Caspase 9, apoptosis-related cysteine peptidase		CASP9-F	AGGCCCCATATGATCGAGGA	193
				CASP9-R	TCGACAACTTTGCTGCTTGC	
NM_001252	CD70	CD70 molecule		CD70-F	GTCACTTGGGTGGGACGTAG	146
				CD70-R	GATGGATACGTAGCTGCCCC	
NM_014430	CIDEB	Cell death-inducing DFFA-like effector b		CIDEB-F	GTCTTCCAGCTCAGCAGTGT	134
				CIDEB-R	AATCCCTGCTTTCCTGCCAA	
NM_019887	DIABLO	Diablo, IAP-binding mitochondrial protein		DIABLO-F	CGTGGCTATGGGGCGAG	189
				DIABLO-R	AGCCACAACAGGAACACACA	

NM_000639	FASLG	Fas ligand (TNF superfamily, member 6)		FAS-F	GTCTACCAGCCAGATGCACA	139
				FAS-R	CCATTCCAGAGGCATGGACC	
NM_000595	LTA	Lymphotoxin alpha (TNF superfamily, member 1)		LTA-F	TGCTTTGGACTACCGCCC	134
				LTA-R	GAAGAGACGTTCAGGTGGTGT	
NM_003844	TNFRSF10A	Tumor necrosis factor receptor superfamily, member 10a		TNFRSF10A-F	CTACCTCCATGGGACAGCAC	162
				TNFRSF10A-R	TTGCAGCTGAGCTAGGTACG	
NM_003842	TNFRSF10B	Tumor necrosis factor receptor superfamily, member 10b		TNFRSF10B-F	GTGTCAGTGCGAAGAAGGCA	163
				TNFRSF10B-R	TTCCCCACTGTGCTTTGTACC	
NM_002546	TNFRSF11B	Tumor necrosis factor receptor superfamily, member 11b		TNFRSF11B-F	GCCTGGCACCAAAGTAAACG	200
				TNFRSF11B-R	GCTCGAAGGTGAGGTTAGCA	
NM_001065	TNFRSF1A	Tumor necrosis factor receptor superfamily, member 1A		TNFRSF1A-F	CCTCAACTGTCACCCCAAGG	190
				TNFRSF1A-R	ATTCCCACCAACAGCTCCAG	
NM_001066	TNFRSF1B	Tumor necrosis factor receptor superfamily, member 1B		TNFRSF1B-F	CACATGCCGGCTCAGAGAAT	144
				TNFRSF1B-R	AGCTGGGTGTATGTGCTGTC	
NM_014452	TNFRSF21	Tumor necrosis factor receptor superfamily, member 21		TNFRSF21 -F	GGGCTTCTTCGTGGATGAGT	199
				TNFRSF21 -R	CCGCAGCTCCTCAGGATTTA	
NM_003790	TNFRSF25	Tumor necrosis factor receptor superfamily, member 25		TNFRSF25-F	CCACACACGGCTACTCTGTT	156
				TNFRSF25-R	AACATCTGCCTCCAGCCAC	
NM_001561	TNFRSF9	Tumor necrosis factor receptor superfamily, member 9		TNFRSF9 -F	CTCACGCTCCGTTTCTCTGT	121
				TNFRSF9 -R	GAAATCGGCAGCTACAGCCA	
NM_003810	TNFSF10	Tumor necrosis factor (ligand) superfamily, member 10		TNFSF10-F	TTGGGACCCCAATGACGAAG	197
				TNFSF10-R	TGGTCCCAGTTATGTGAGCTG	
NM_003789	TRADD	TNFRSF1A-associated via death domain		TRADD-F	GAAGCGGCGGAGTAGAGC	139
				TRADD-R	AGCGAGGACTCCACAAACAG	
NM_021138	TRAF2	TNF receptor-associated factor 2		TRAF2-F	TCCCCTTAACTTGTGACGGC	105
				TRAF2-R	CGTGGAATCTGCAAGGGACT	
NM_005163	AKT1	V-akt murine thymoma viral oncogene homolog 1	Anti-Apoptotic	AKT1-F	CAGGATGTGGACCAACGTGA	137
				AKT1-R	AAGGTGCGTTCGATGACAGT	
NM_004323	BAG1	BCL2-associated athanogene		BAG1-F	ACCGTTGTCAGCACTTGGAA	198
				BAG1-R	GCTTGCAAATCCTTGGGCAG	
NM_003921	BCL10	B-cell CLL/lymphoma 10		BCL10 -F	GGTCTGGACACCCTTGTTGA	155
				BCL10 -R	GCTCCATCTGGAAAAGGTTCAC	
NM_000633	BCL2	B-cell CLL/lymphoma 2		BCL2-F	CTTTGAGTTCGGTGGGGTCA	162
				BCL2-R	GGGCCGTACAGTTCCACAAA	
NM_004049	BCL2A1	BCL2-related protein A1		BCL2A1-F	GATAAGGCAAAACGGAGGCTG	133
				BCL2A1-R	TGGTCAACAGTATTGCTTCAGGA	
NM_016561	BFAR	Bifunctional apoptosis regulator		BFAR-F	TTTAGCTCCTAACACAGGCCG	178
				BFAR-R	AACTTCTTCCGCCGTCCATT	
NM_001242	CD27	CD27 molecule		CD27-F	TGCCAGATGTGTGAGCCAG	191
				CD27-R	TCAGCATTGGCAGTGATGGT	

NM_001250	CD40	CD40 molecule, TNF receptor superfamily member 5		CD40-F	GGCAGGCACAAACAAGACTG	139
				Cd40-R	TGGCTTCTTGGCCACCTTTT	
NM_000572	IL10	Interleukin 10		IL10-F	GGGCACCCAGTCTGAGAAC	190
				IL10-R	CTCAGACAAGGCTTGGCAAC	
NM_003946	NOL3	Nucleolar protein 3 (apoptosis repressor with CARD domain)		NOL3-F	TCTGGAGAGGCAGGAGGAC	137
				NOL3-R	CTCGCGGTCGATAGTCTCTG	
NM_003998	NFKB1	Nuclear factor of kappa light polypeptide gene enhancer in B-cells 1		NFKB1-F	GCTTAGGAGGGAGAGCCCA	196
				NFKB1-R	GGTATGGGCCATCTGCTGTT	
NM_004330	BNIP2	BCL2/adenovirus E1B 19kDa interacting protein 2		BNIP2-F	CCTGCTTTTTGCGACCTGG	103
				BNIP2-R	AATCCAGGGAGCCAATGTCC	
NM_001165	BIRC3	Baculoviral IAP repeat containing 3		BIRC3-F	GCTTGCAAGTGCGGGTTTTTA	185
				BIRC3-R	TGGCTTGAACTTGACGGATGA	
NM_004536	NAIP	NLR family, apoptosis inhibitory protein		NAIP-F	ATTGGGGACTTCGTCTGGGA	183
				NAIP-R	GGGTGGCCATTTTCTGAGGT	
NM_001101	ACTB	Actin, beta	Reference	ACTB-F	AGAAAATCTGGCACCACACCT	187
				ACTB-R	GAGGCGTACAGGGATAGCAC	
NM_002046	GAPDH	Glyceraldehyde-3-phosphate dehydrogenase		GAPDH-F	GGAAGGTGAAGGTCGGAGTC	108
				GAPDH-R	TGAAGGGGTCATTGATGGCA	
