# Supplementary figures and images for: Galactosylceramide Upregulates the Expression of the BCL2 Gene and Downregulates the Expression of TNFRSF1B and TNFRSF9 Genes, Acting as an Anti-Apoptotic Molecule in Breast Cancer Cells
Source: Cancers (Basel). 2024 Jan 17;16(2):389. doi: 10.3390/cancers16020389 (PMC10813928; doi:10.3390/cancers16020389)

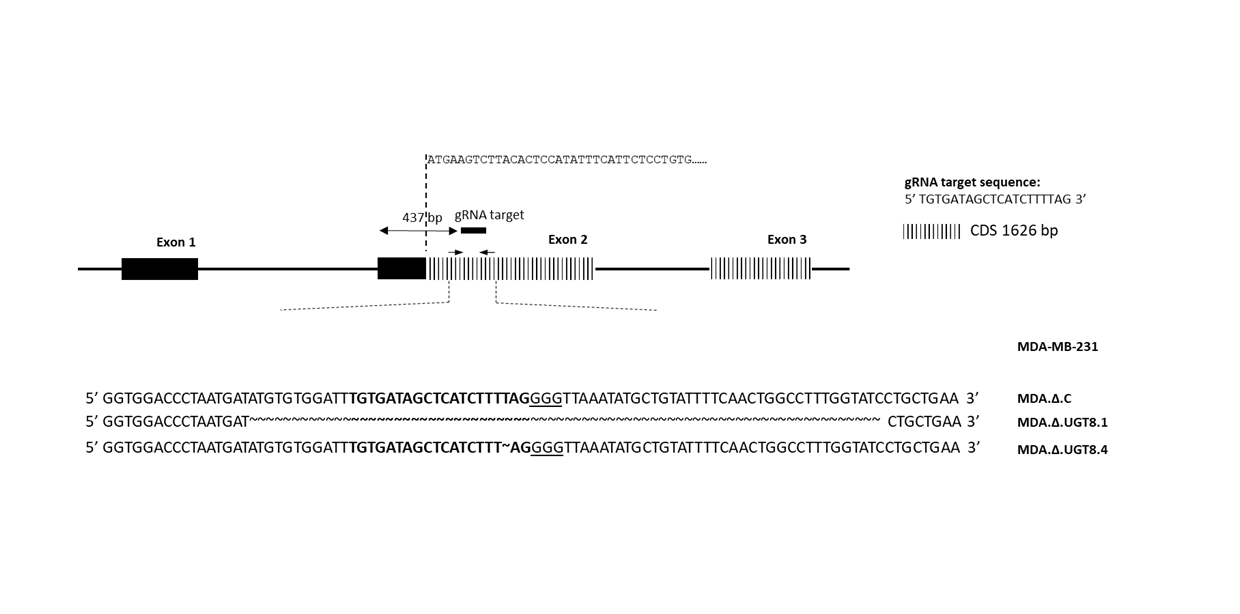

Supplement: Supplementary file 1 [file cancers-16-00389-s001.zip › Fig S1.tif]

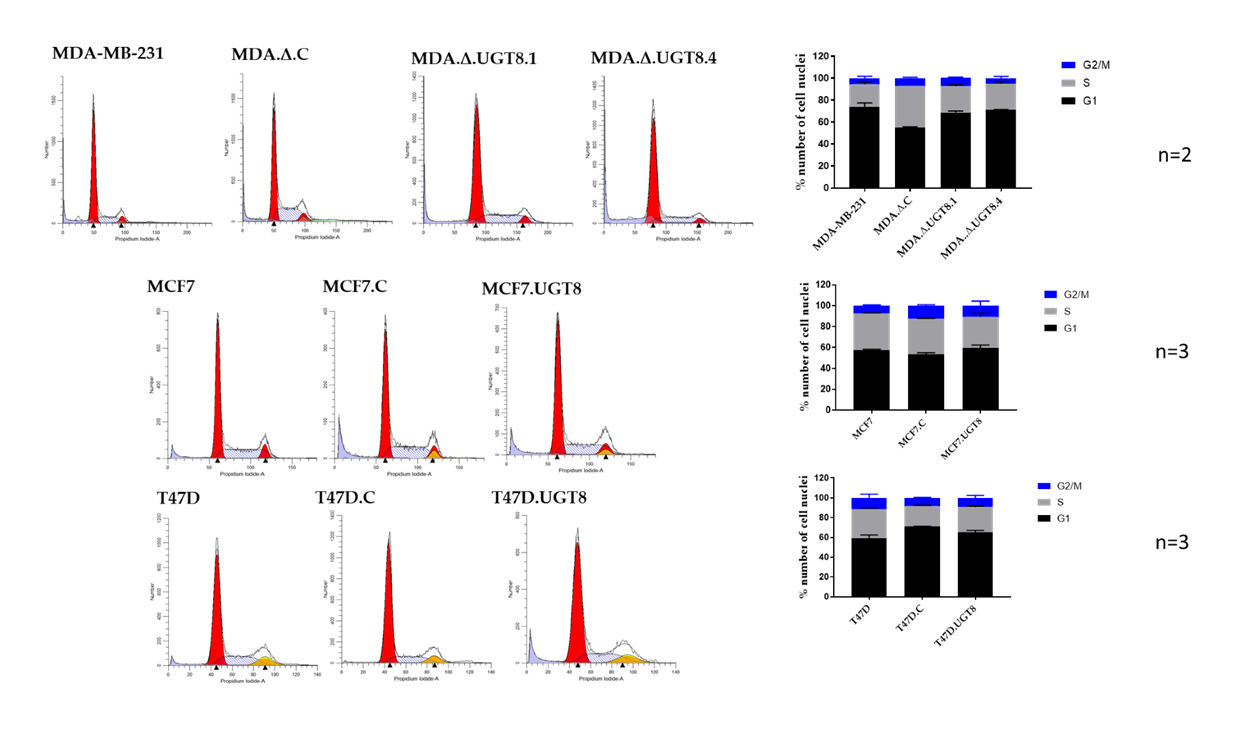

Supplement: Supplementary file 1 [file cancers-16-00389-s001.zip › Fig S2.tif]

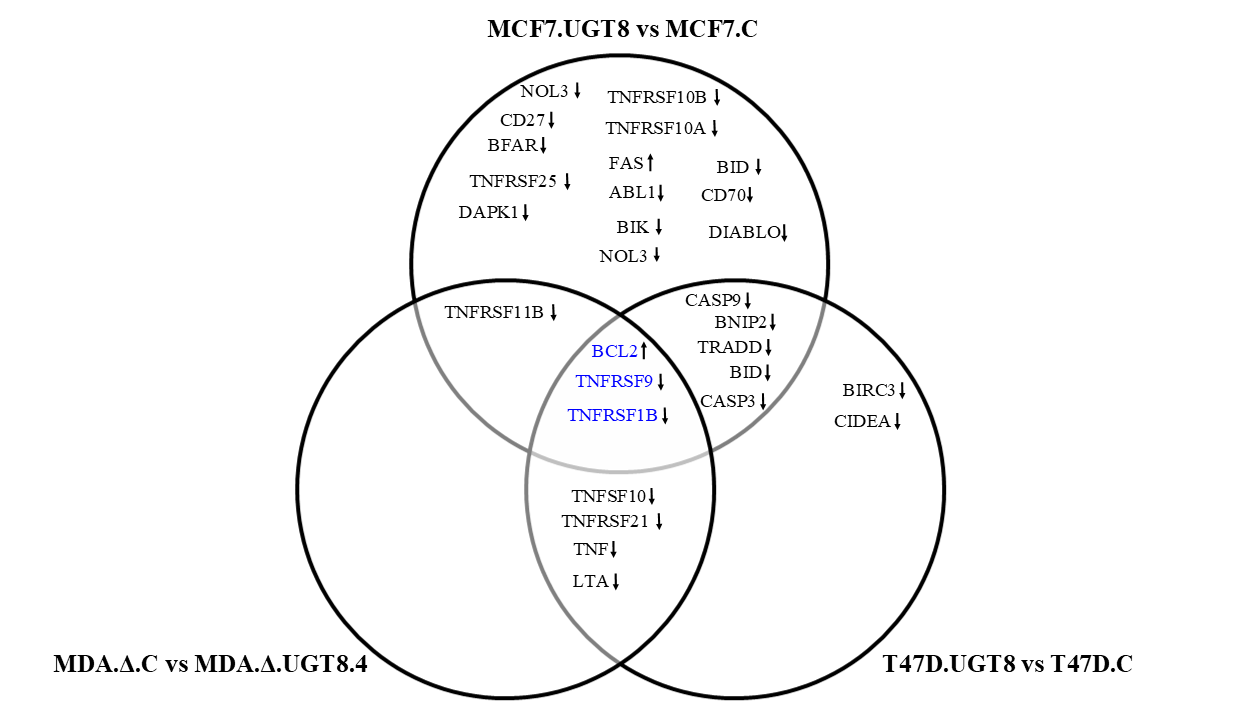

Supplement: Supplementary file 1 [file cancers-16-00389-s001.zip › Fig S3.tif]

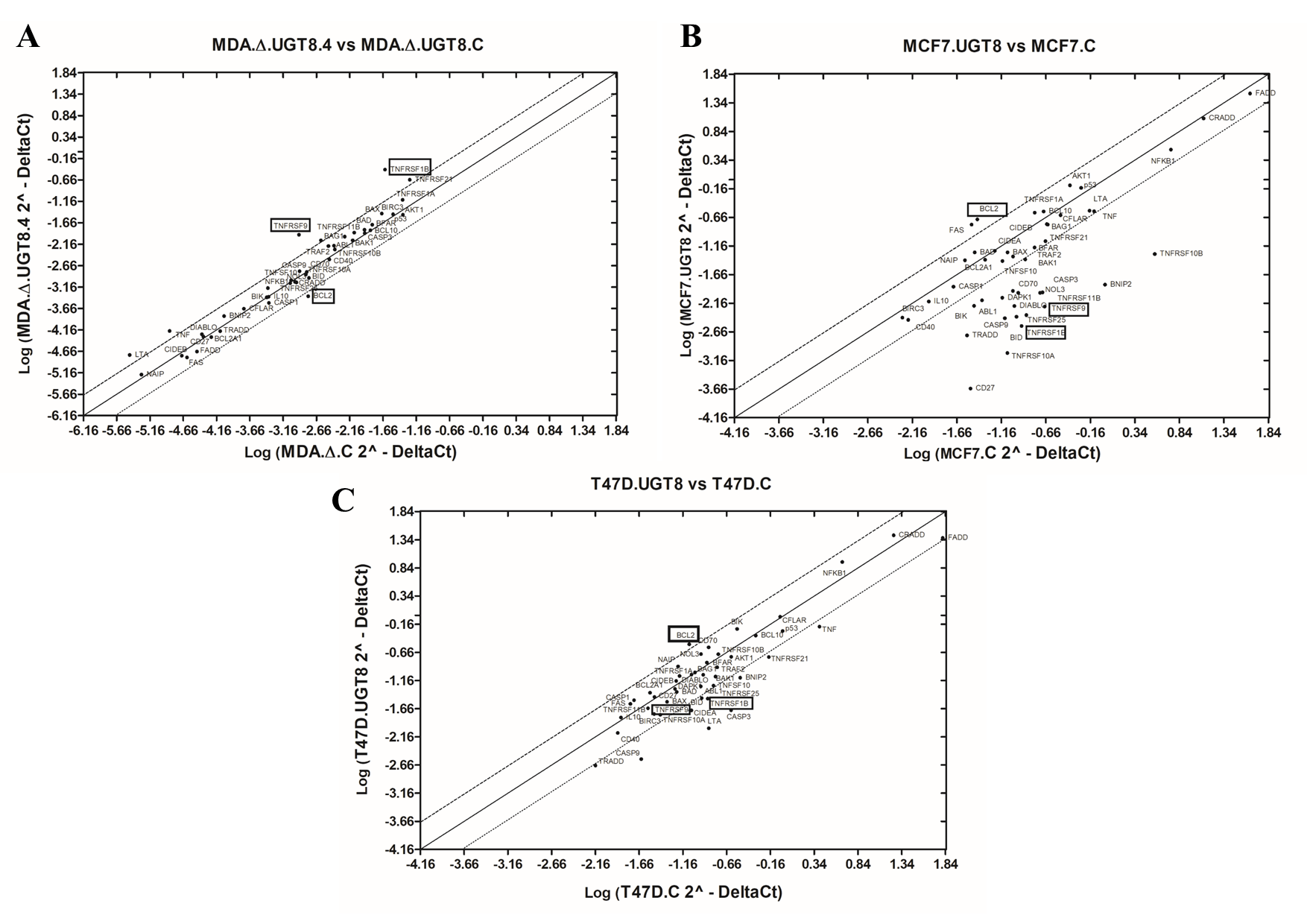

Supplement: Supplementary file 1 [file cancers-16-00389-s001.zip › Fig S4.tif]

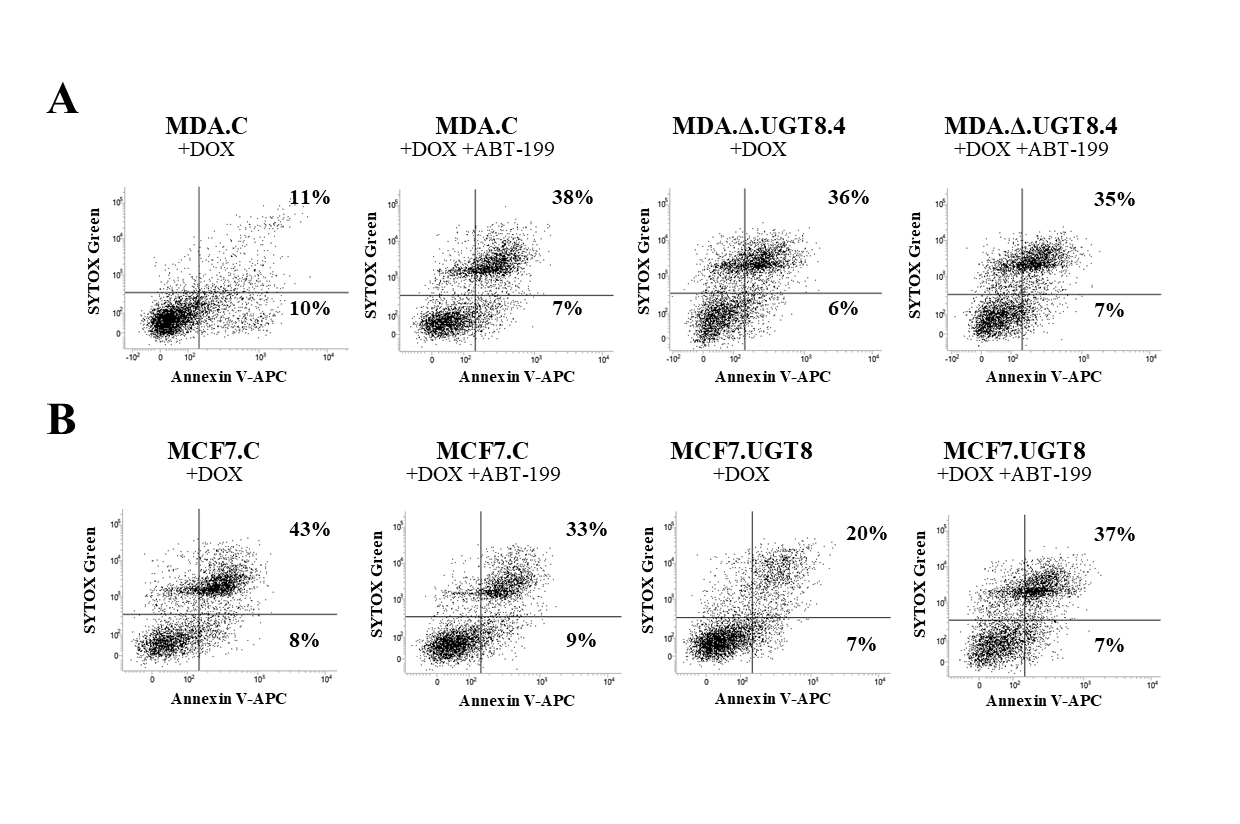

Supplement: Supplementary file 1 [file cancers-16-00389-s001.zip › Fig S5.tif]

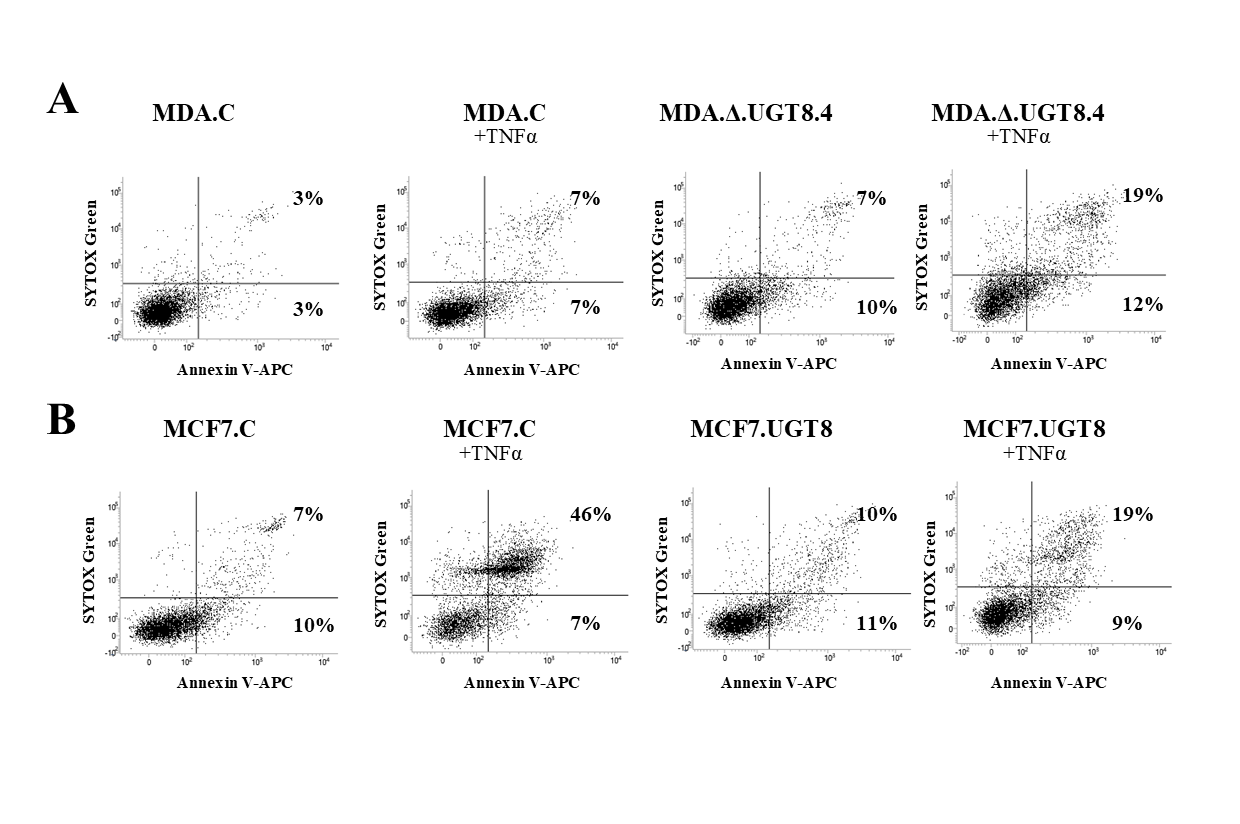

Supplement: Supplementary file 1 [file cancers-16-00389-s001.zip › Fig S6.tif]

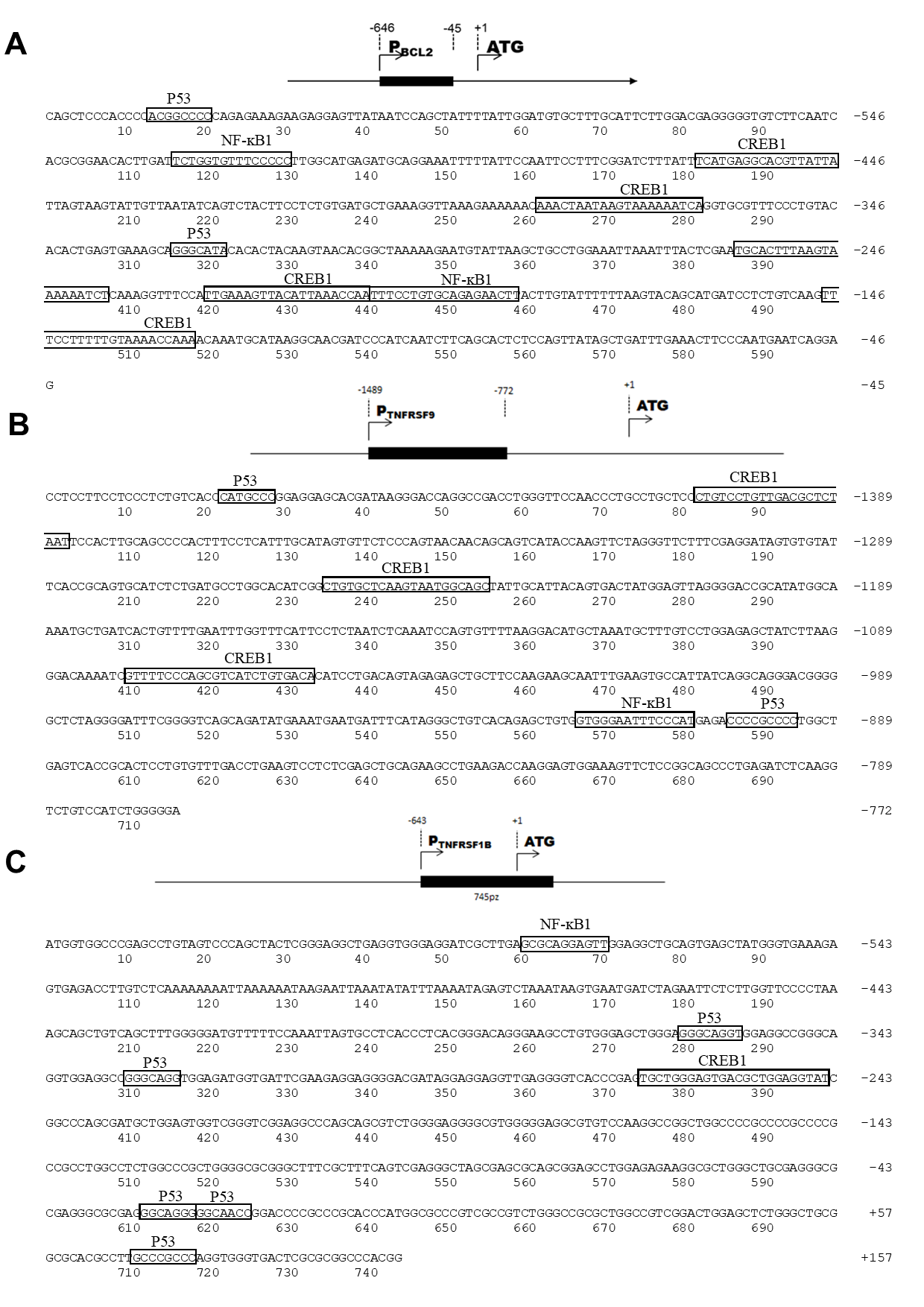

Supplement: Supplementary file 1 [file cancers-16-00389-s001.zip › Fig S7.tif]

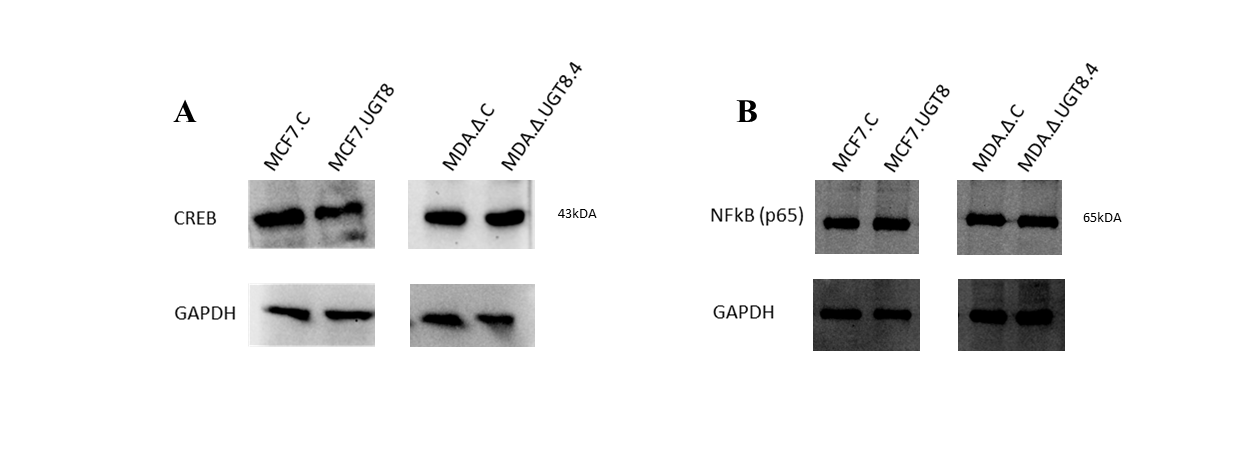

Supplement: Supplementary file 1 [file cancers-16-00389-s001.zip › Fig S8.tif]

Fig 1

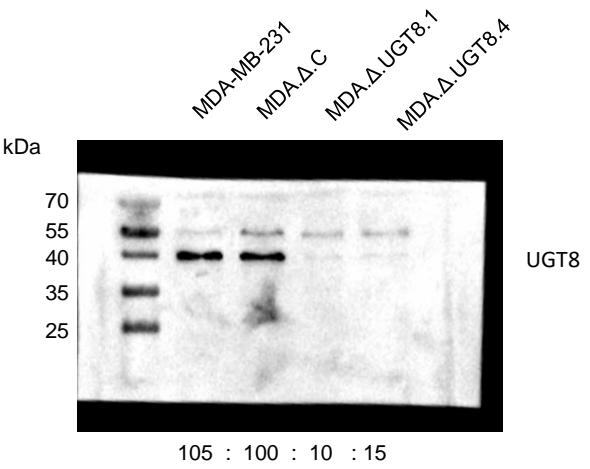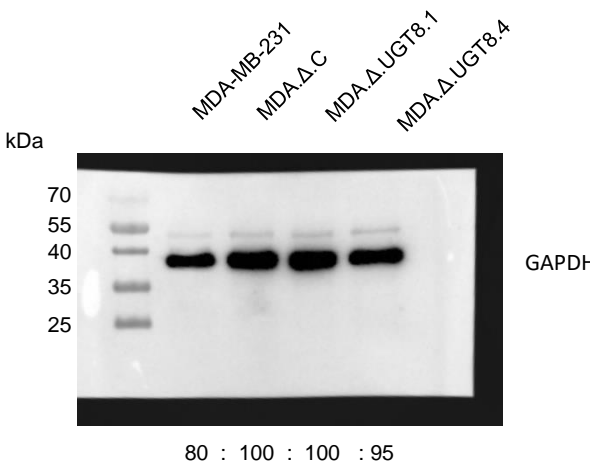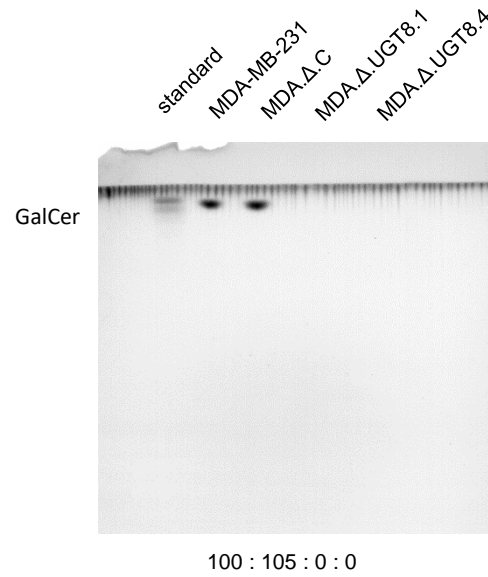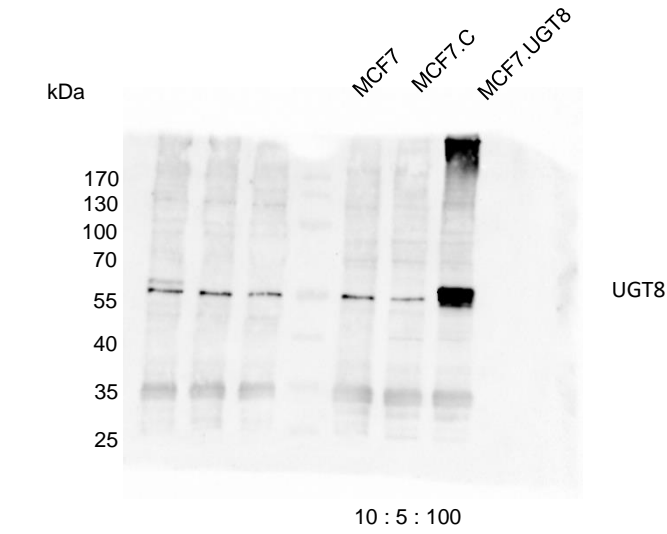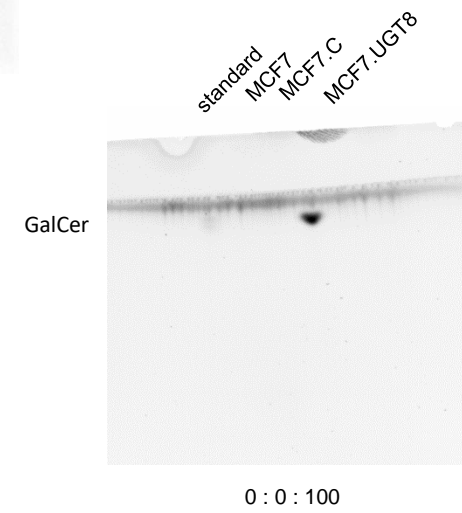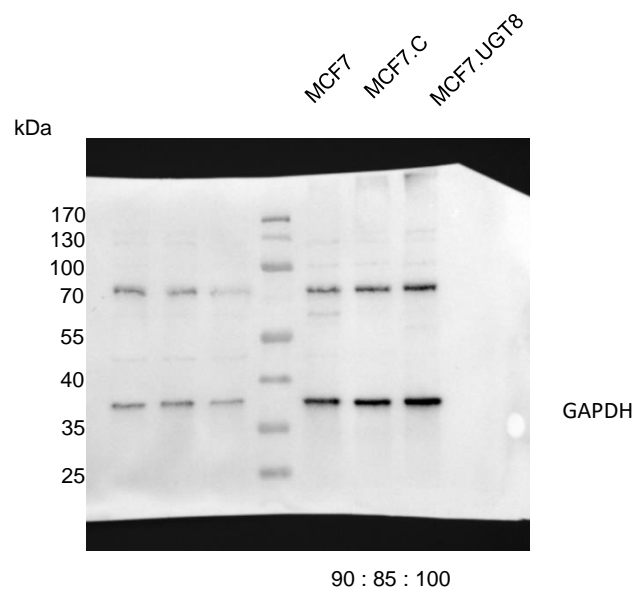

Fig 2

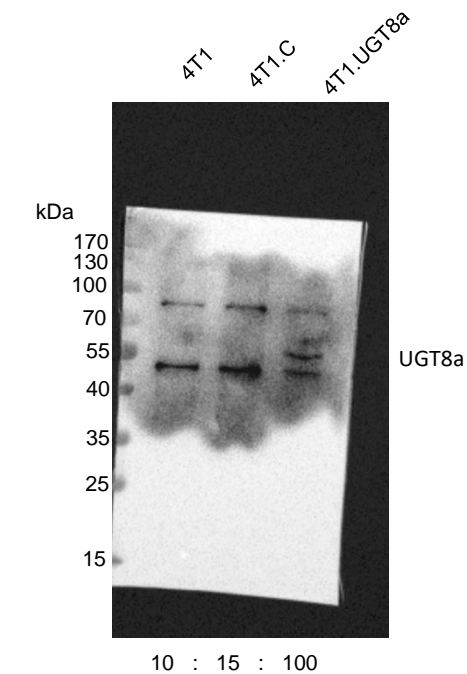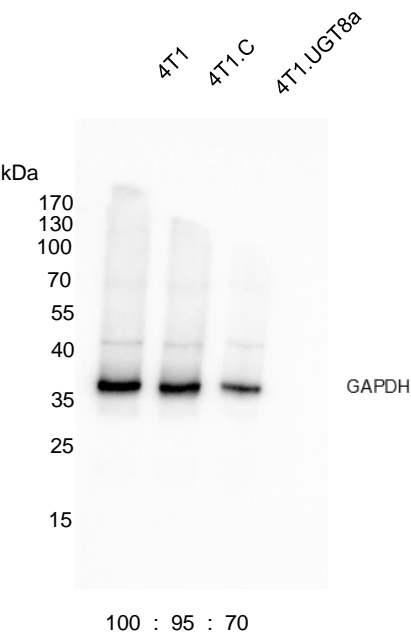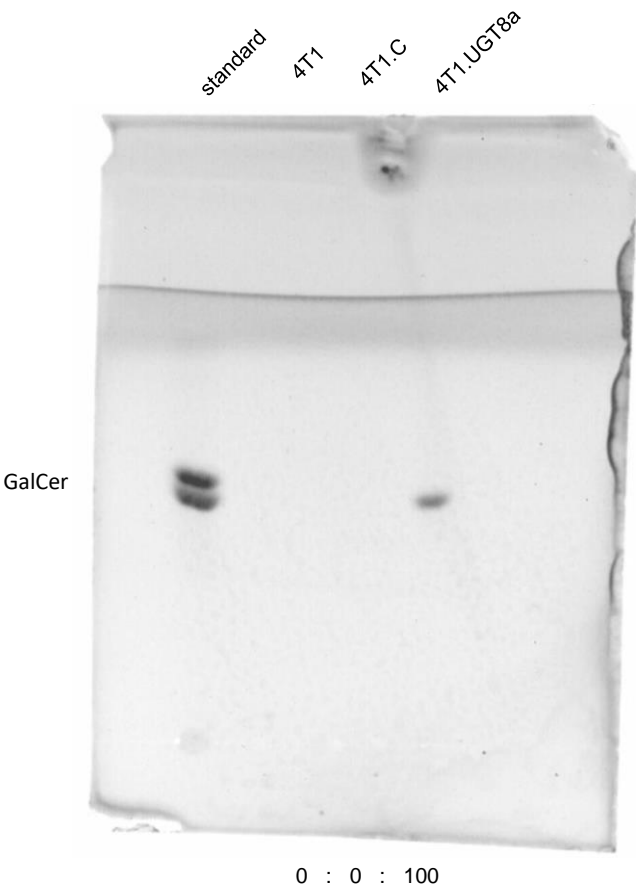

Fig 3B

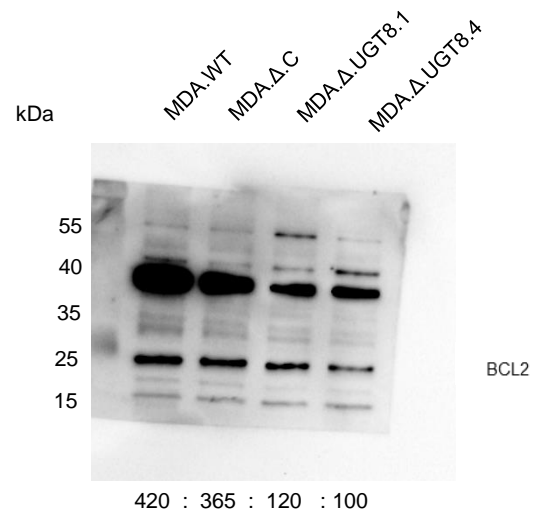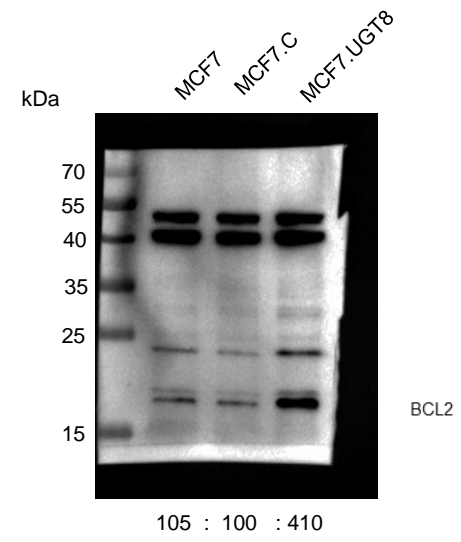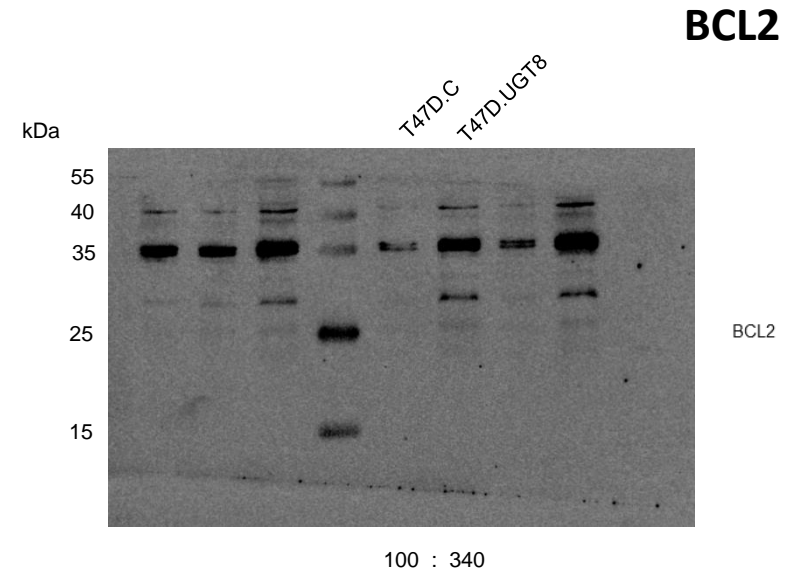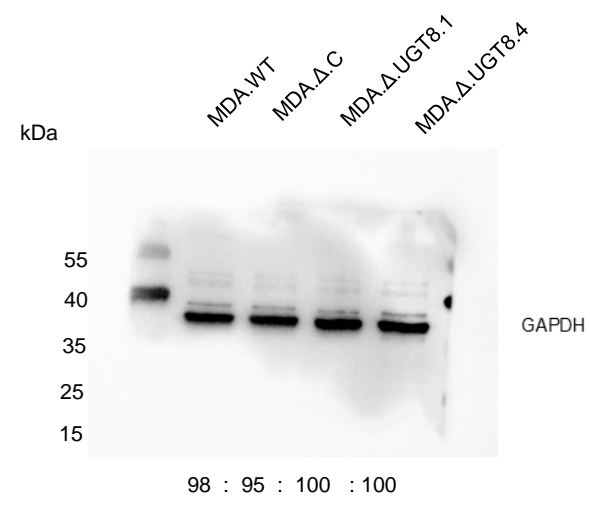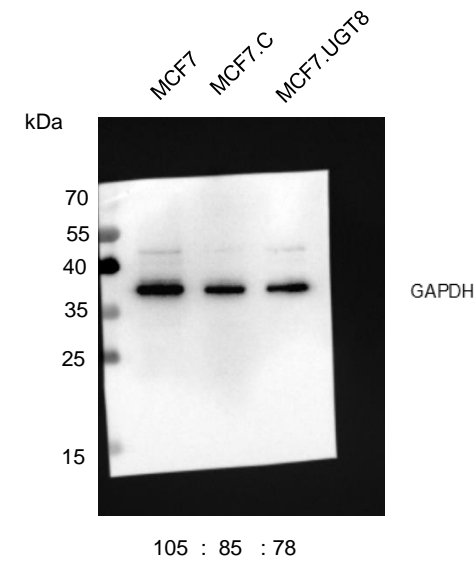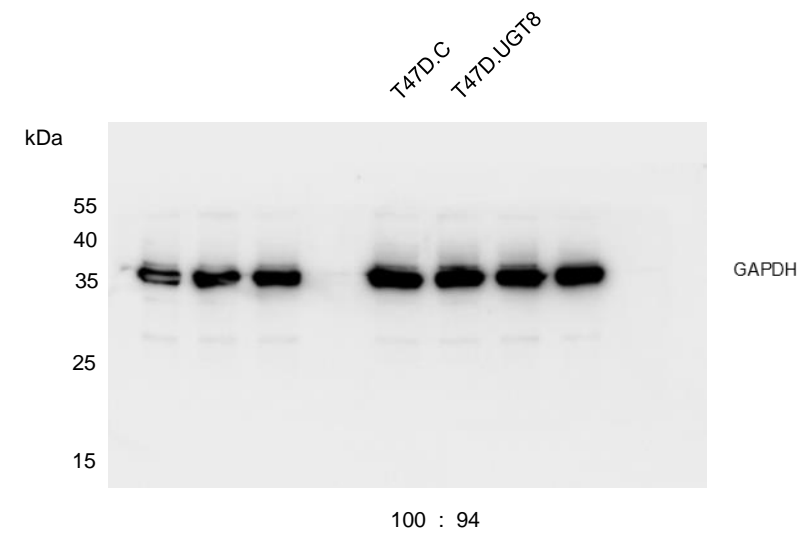

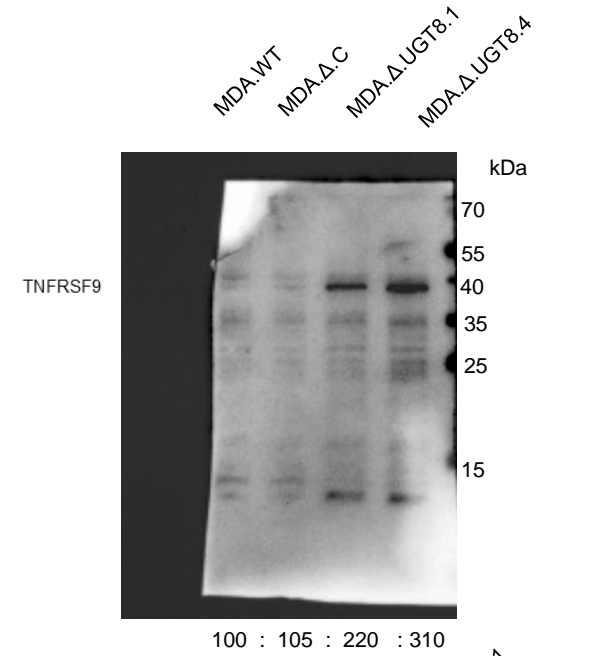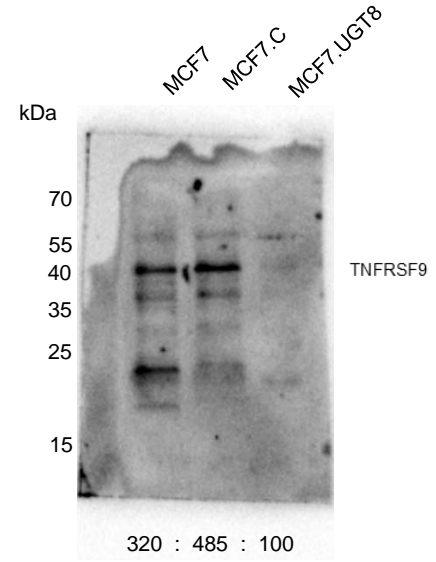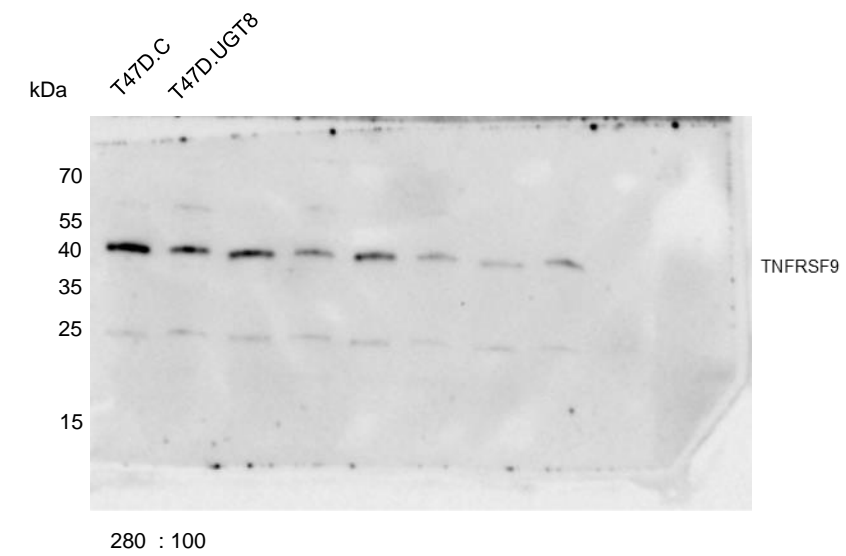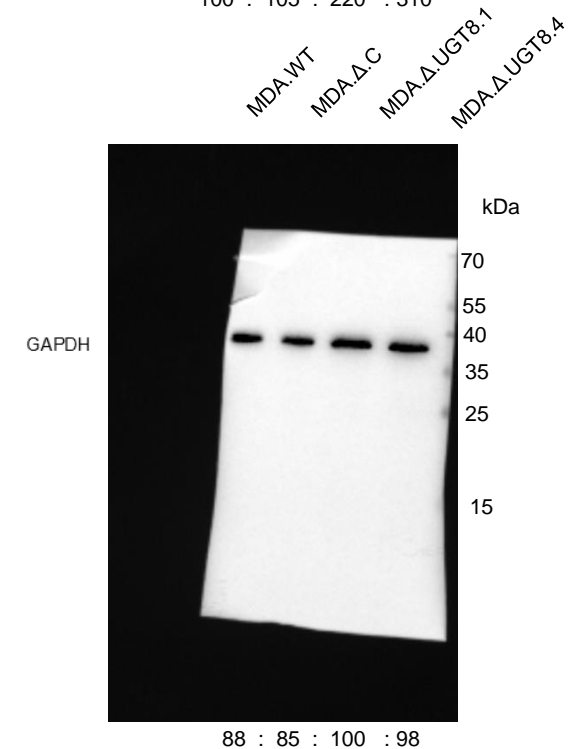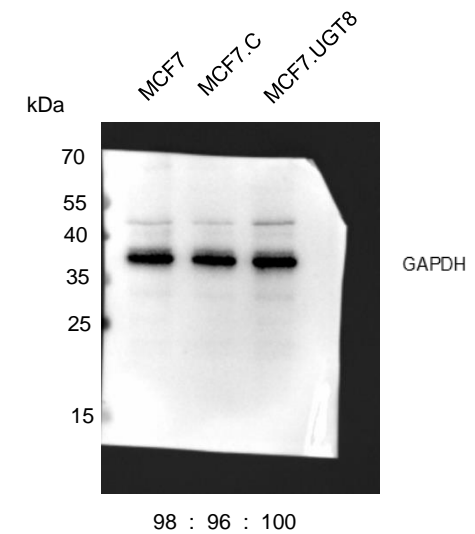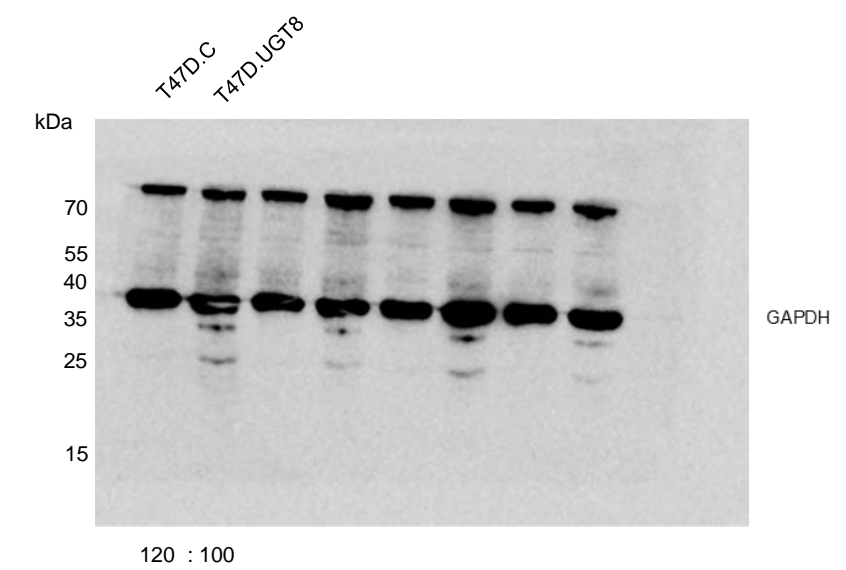

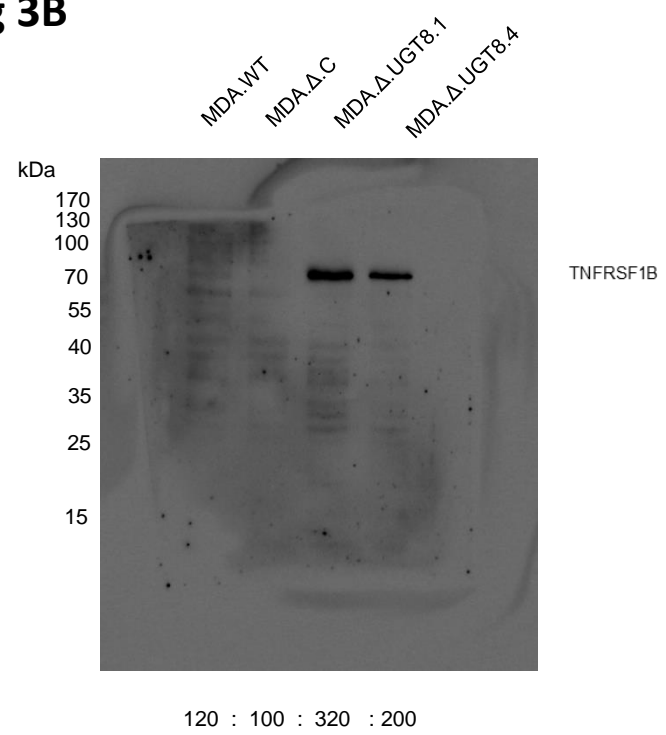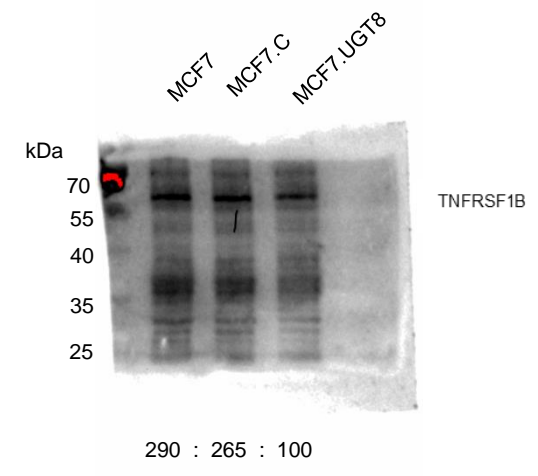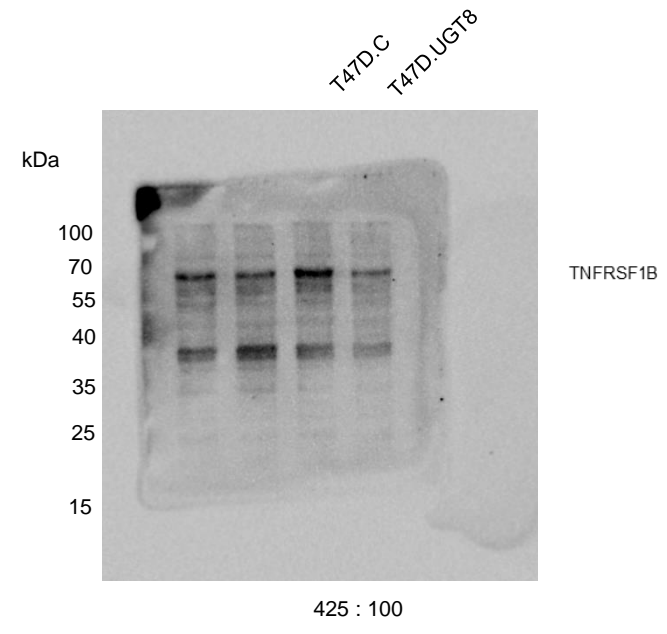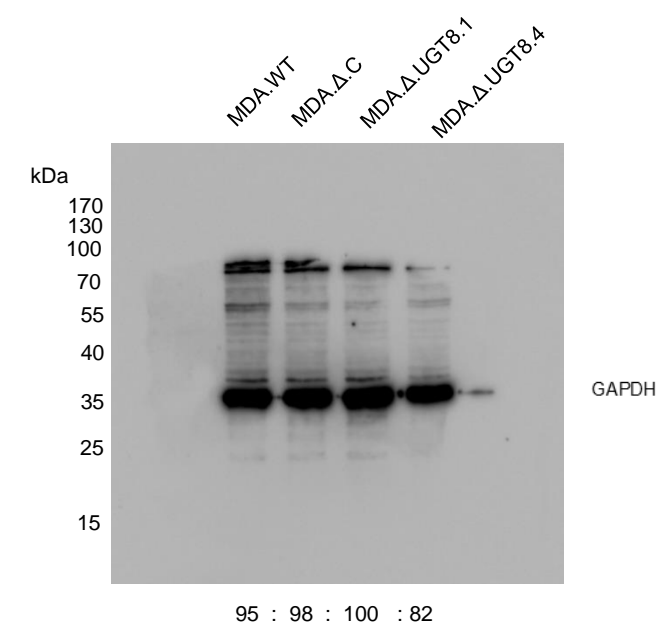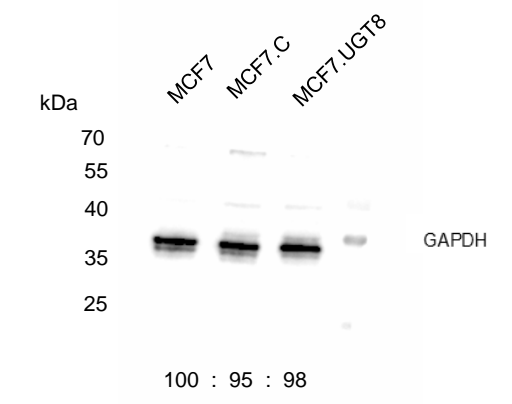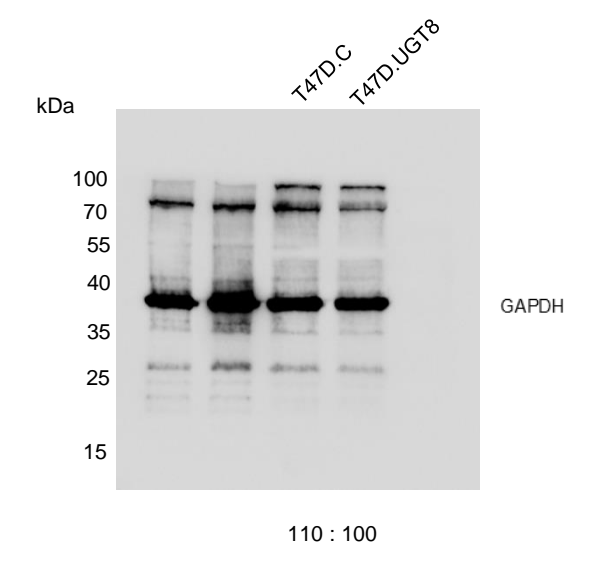

Fig 6A

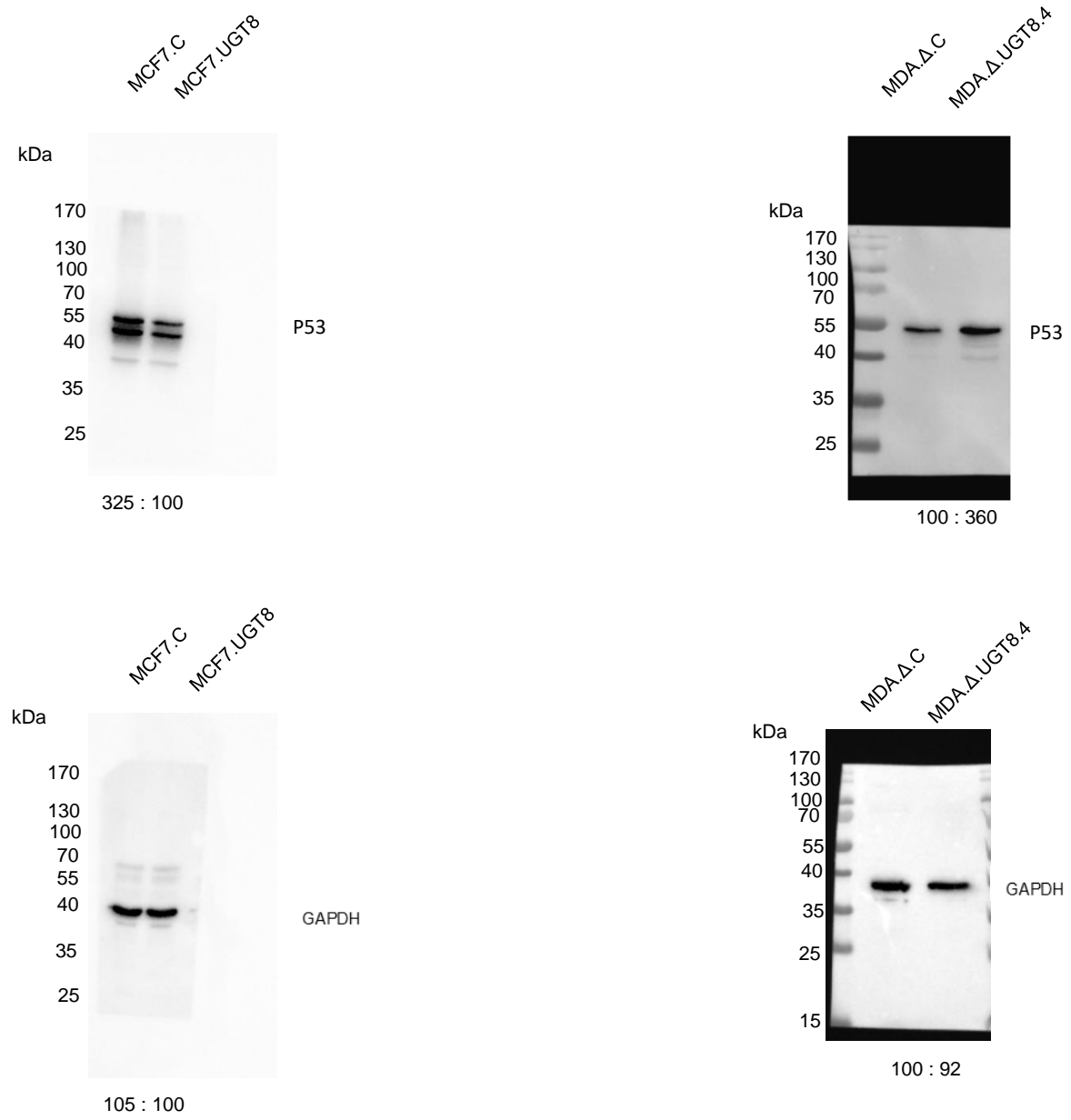

Sup. Fig S8

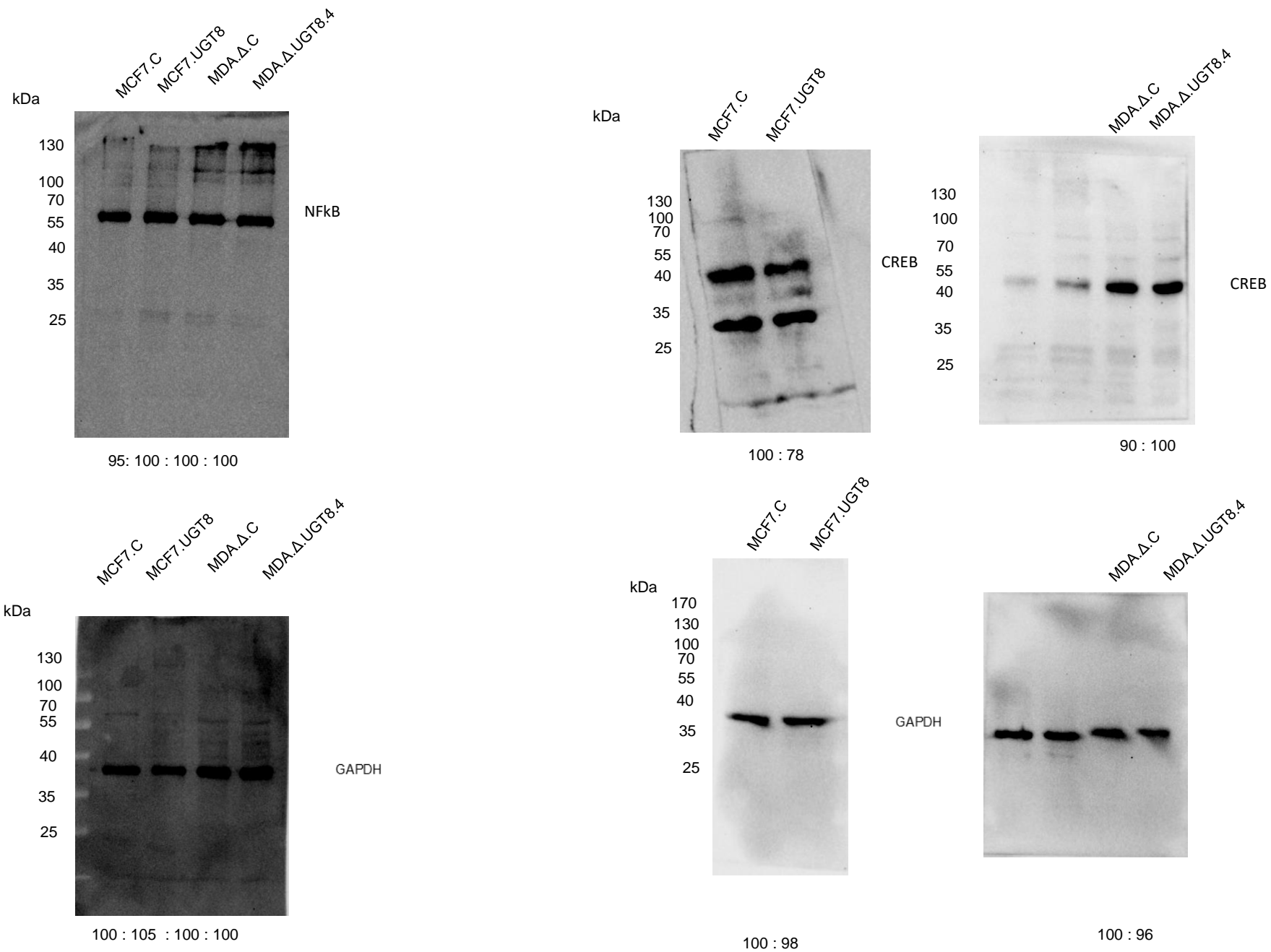

Supplement: Supplementary file 1 [file cancers-16-00389-s001.zip › Figure S9.pdf]
